# Evidence-Based Analysis of AI Chatbots in Oncology Patient Education: Implications for Trust, Perceived Realness, and Misinformation Management

**DOI:** 10.1007/s13187-025-02592-4

**Published:** 2025-02-18

**Authors:** Aaron Lawson McLean, Vagelis Hristidis

**Affiliations:** 1 Department of Neurosurgery, Jena University Hospital – Friedrich Schiller University Jena, Am Klinikum 1, 07747 Jena, Germany; 2 Comprehensive Cancer Center Central Germany, Jena, Germany; 3 Computer Science and Engineering, University of California, Riverside, Riverside, CA USA

**Keywords:** Artificial intelligence, Chatbots, Oncology education, Cancer information, Misinformation control, Patient empowerment

## Abstract

The rapid integration of AI-driven chatbots into oncology education represents both a transformative opportunity and a critical challenge. These systems, powered by advanced language models, can deliver personalized, real-time cancer information to patients, caregivers, and clinicians, bridging gaps in access and availability. However, their ability to convincingly mimic human-like conversation raises pressing concerns regarding misinformation, trust, and their overall effectiveness in digital health communication. This review examines the dual-edged role of AI chatbots, exploring their capacity to support patient education and alleviate clinical burdens, while highlighting the risks of lack of or inadequate algorithmic opacity (i.e., the inability to see the data and reasoning used to make a decision, which hinders appropriate future action), false information, and the ethical dilemmas posed by human-seeming AI entities. Strategies to mitigate these risks include robust oversight, transparent algorithmic development, and alignment with evidence-based oncology protocols. Ultimately, the responsible deployment of AI chatbots requires a commitment to safeguarding the core values of evidence-based practice, patient trust, and human-centered care.

## Introduction

Recent advancements in conversational artificial intelligence (AI) have empowered technology firms to create sophisticated chatbots capable of replicating human-like writing and speech patterns, emotional nuances, and even presumed backstories of real people [1, 2]. In oncology education, these AI-driven tools, whether text-based or voice-enabled, promise to offer instant, on-demand guidance for patients and caregivers, circumventing time and resource constraints [3]. Yet they also raise challenging questions about authenticity, accuracy, and the potential erosion of trust in legitimate sources of cancer information. Indeed, the rising prevalence of “human-seeming” chat agents, especially on high-traffic platforms like those operated by Meta, underscores how easily the lines between human expertise and algorithmic mimicry can blur [4].

A key factor contributing to the perception of large language models (LLMs) is their ability to produce grammatically correct and semantically coherent sentences, often using eloquent and sophisticated language. However, their functional and reasoning capabilities remain limited. Recent research highlights that while LLMs excel in formal competence—producing text that appears linguistically polished—their performance on tasks requiring deeper functional understanding is significantly weaker [5].

This disparity is further complicated by the tendency of individuals to equate language proficiency with intelligence, leading many to assume that LLM-generated responses are accurate. Identifying errors in these responses often requires considerable effort, even for experts. The challenge is amplified when advanced voice-generation tools are used to convert LLM outputs into speech that sounds natural and human-like, potentially resembling the voice of a trusted healthcare professional. This combination of linguistic fluency and lifelike voice delivery heightens the risk of users misplacing trust in these systems. The result is a scenario in which chatbots may convincingly deliver erroneous advice, conceal their non-human identity, or perpetuate misinformation about screening procedures, newly approved drugs, or palliative care.

Overlaying these immediate risks is the so-called Dead Internet Theory, which posits that a substantial fraction of online traffic may originate from automated bots rather than actual humans. Although widely debated, the broad premise has found new resonance in the public sphere, fueled by stories of entire networks of automated users interacting with one another and shaping online discourse [6]. In oncology education, a domain where accurate, empathetic communication is often critical, the specter of a “dead internet” becomes problematic if it undermines confidence in digital tools that have, in many cases, been extremely useful in disseminating scientific findings and supporting patients. This review examines how “human-seeming” AI chatbots might fit into this fraught landscape, outlines both potential benefits and emerging hazards, and proposes possible solutions to ensure that the future of cancer education remains firmly grounded in best practices.

## Background on AI Chatbots and the Illusion of Realness

LLMs have evolved quickly, moving from rudimentary question–answer capabilities to highly adaptive systems that produce content nearly indistinguishable from that written by humans [7, 8]. This enhanced fluency has made them attractive for a multitude of tasks, including triaging medical inquiries, explaining treatment guidelines, and providing emotional support to individuals struggling with cancer diagnoses [9, 10]. One perceived advantage is the chatbot’s ability to provide round-the-clock assistance at scale, reaching individuals who might not have ready access to oncologists or specialized clinics. Yet the same linguistic sophistication that facilitates broad outreach can also create illusions of authority and empathy, enticing users to rely on a “helpful online confidant” that is ultimately an algorithm [11, 12].

In cancer care, trust and verifiability are paramount. Patients confronting life-changing decisions depend on detailed conversations with oncologists, nurses, and counselors whose credentials, licensing, and experience are documented and transparent [13]. A chatbot’s “realness”—that is, the extent to which it can replicate the subtle cues of human conversation—can short-circuit this dynamic. Users might form attachments to the chatbot, perceiving it as a trusted “partner,” only to learn that it is driven by statistical pattern recognition, not medical judgment [14, 15]. The problem is further compounded by the fact that many chatbots are developed by proprietary firms—privately owned companies that tightly control their software, data, and development processes—while withholding details about their training data and modeling techniques [16]. It therefore becomes unclear whether these systems are anchored in reputable oncology guidelines or gleaning their statements from unverified blog posts and tangential online sources.

When one factors in initiatives like Meta’s recent announcement to embed AI “personas” into social media platforms, the idea of encountering entire clusters of fictional digital “entities” masquerading as real people is no longer speculative [17]. These hyper-realistic chatbots can share personal stories of “surviving cancer,” comment on new therapies, or even console frightened users seeking help. While such interactions might be well intentioned, if, for instance, they provide accurate resources or promote screening, they could just as easily spread misguided healthcare advice. The underlying tension is heightened by reports that some AI chatbots have made spurious references to clinical trials, non-existent peer-reviewed studies, or even contrived personal anecdotes [18]. In a domain as sensitive as oncology, such deceptions may magnify patient anxiety, divert people from recommended care, or diminish credibility of legitimate cancer-support communities [19]. The issue of generating false or imaginary information is not limited to healthcare; AI systems have also fabricated legal cases, further demonstrating the potential risks of deploying chatbots without stringent safeguards [20].

### Connecting Human-Seeming Chat Partners and the “Dead Internet Theory”

The “Dead Internet Theory” suggests that a meaningful fraction of what appears to be organic online engagement (i.e., genuine interactions between real human users on digital platforms) is actually automated, orchestrated by advanced bots [21]. While much of this notion remains speculative, it resonates with concerns that the internet’s once-vibrant human exchange could be drowned out, or even replaced, by mechanized systems. If indeed large swaths of social media users, commentators, and “supporters” in cancer forums are bots, the concept of a robust, empathetic online community becomes precarious. The presence of AI-driven cancer chatbots that convincingly pass as genuine survivors or dedicated volunteers effectively reinforces this skepticism [22].

Should it turn out that many helpful “voices” in online oncology groups are non-human, the psychological consequences for genuine participants may be profound. Patients may feel alienated if they realize that the words of comfort or personal experiences come from a language model, rather than someone who truly walked that journey. Others might lose confidence in digital channels for health advice altogether, creating a ripple effect in communities where reputable telemedicine or genuine patient-led advocacy has been a lifeline [23]. In this sense, the interplay between the “dead internet” hypothesis and human-seeming chat partners raises broader questions about how best to preserve authenticity and ensure that technology enhances, rather than compromises, the human dimension of cancer care.

Another significant concern is the risk of training recursion, which occurs when LLMs increasingly rely on data generated by other LLMs rather than human-produced content. This feedback loop has been shown to degrade the performance and accuracy of LLMs over time, as the quality and diversity of the training data diminish [24].

### Current Evidence of Chatbot Integration in Oncology Education

Despite these concerns, AI chatbots can confer certain benefits, particularly in educational contexts [25–28]. When powered by meticulously curated data sets and regularly monitored by expert clinicians, chatbots are capable of answering straightforward questions about cancer biology, standard therapies, and the logistics of appointments or follow-up visits. Studies exploring their utility suggest that patients value the immediacy of responses, the ability to pose questions multiple times without feeling guilty, and the non-judgmental tone. In these investigations, participants reported improved recall of basic information about side effects and were more likely to adhere to recommended screening intervals [29–33].

However, the distinction between a beneficial chatbot and one that might cause confusion or harm is not always evident. Many systems lack robust transparency mechanisms to declare, prominently and repeatedly, that they are computer programs rather than living counselors or clinicians. Often, disclaimers exist but are buried in terms-of-service documents or at the bottom of a chat window, insufficient to prevent users from concluding that they are conversing with a knowledgeable individual. Early-phase evaluations of “human-approximate” chatbots in oncology have highlighted this divergence between user perception and the system’s true nature [34, 35]. Some chatbots have included well-intentioned emotional content in their answers, for example, responding “I’m sorry to hear that” or “I know how frightening it can be.” While such empathetic phrases can be comforting, they can also give patients a misleading sense of personal understanding and clinical acumen on the part of the chatbot.

Chatbot guardrails are widely utilized to constrain the scope of chatbot responses, ensuring they remain within predefined boundaries. These mechanisms have proven effective in restricting outputs on sensitive topics, such as political opinions or other controversial subjects, in commercial chatbot applications [36]. Applying similar guardrails to oncology chatbots could help ensure that their responses are aligned with evidence-based guidelines and remain focused on providing accurate, relevant healthcare information.

Researchers evaluating pilot chatbot deployments in cancer education have found a surprisingly high level of user engagement, suggesting that the line between curiosity-driven usage and genuine reliance on AI for decisions may be quite thin [33]. In these scenarios, the underlying algorithms were generally not advanced enough to mislead participants about being human. Yet as LLMs grow more sophisticated, the risk of deception rises, particularly if the chatbot adopts a persona—complete with a name, a fictional biography, and a semblance of shared experiences. This phenomenon disrupts established heuristics about trusting digital sources: historically, users who encountered simplistic automated systems recognized them as mere scripts, whereas present-day technologies are advanced enough to trigger “presence illusions” [37].

### Ethical and Practical Complications

At the heart of the issue lies an ethical paradox: the more closely an AI system mimics a caring, experienced oncology professional or a sympathetic survivor, the more it can manipulate user emotion, wittingly or unwittingly. This deception might arise even if the chatbot’s developer has no malicious intent. The authenticity crisis intensifies in public forums, where these bots could unilaterally reply to posts with questionable or dangerously incomplete information. Under the “dead internet” perspective, a portion of those replies might even come from other AI agents reinforcing or echoing the initial statement, an echo chamber of automated participants, all “talking” to each other and presenting a unified but potentially erroneous message [38].

Among the key hurdles is the challenge of accuracy. Though chatbots can be pre-trained on reputable cancer information, new treatment guidelines or newly published clinical trial data appear at a swift pace, meaning the system must be updated. If periodic re-training processes fail to incorporate the latest oncology standards, or if the chatbots inadvertently integrate unverified content, misinformation will gradually accumulate [39, 40]. The impetus to operate chatbots in real-time on social media platforms further heightens this risk. Continuous, large-scale user interactions might shift the chatbot’s language model weights (in certain adaptive paradigms), making it prone to drifting away from evidence-based knowledge [41]. For example, an oncology chatbot initially programmed with accurate data on chemotherapy protocols may become outdated if it fails to integrate emerging evidence on novel immunotherapies or targeted treatments, potentially leading patients to make uninformed decisions based on obsolete recommendations. Similarly, if a chatbot repeatedly encounters and internalizes user-generated content that misrepresents clinical trial eligibility criteria, such as falsely suggesting that all stage IV cancer patients qualify for experimental therapies, it could unintentionally propagate these inaccuracies, undermining both patient decision-making and clinician guidance.

Beyond accuracy, privacy concerns loom large. Patients discussing personal health matters with a chatbot risk exposing detailed clinical histories, psychological states, or preferences about therapy. The chatbot, if improperly configured, might store or inadvertently reveal these disclosures to third parties, undermining patient confidentiality [42]. More subtly, a generative model that was originally trained on real-world patient records might replicate fragments of those records if prompted in certain ways. This overlap between the chatbot’s structured “memory” and user interactions raises troubling scenarios in which personal information emerges out of context, or in which anonymized data become re-identified through repeated conversation [43].

### The CARE-AI Approach and Emerging Responses

Addressing these concerns calls for initiatives like the proposed CARE-AI (Collaborative Assessment for Responsible and Ethical AI Implementation) framework, which seeks to align AI technologies with rigorous ethical standards and practical safeguards [44]. CARE-AI emphasizes risk assessment for misinformation, data privacy, fairness across diverse patient populations, and transparent declarations of an AI system’s non-human nature. Such a model is adaptable to oncology education, where ensuring the veracity and safety of content is paramount. For instance, a clinical center could mandate that any chatbot integrated into patient education portals undergo third-party evaluations, focusing on the chatbot’s risk of generating harmful advice and its provisions for disclaimers about its automated nature.

**Fig. 1:**
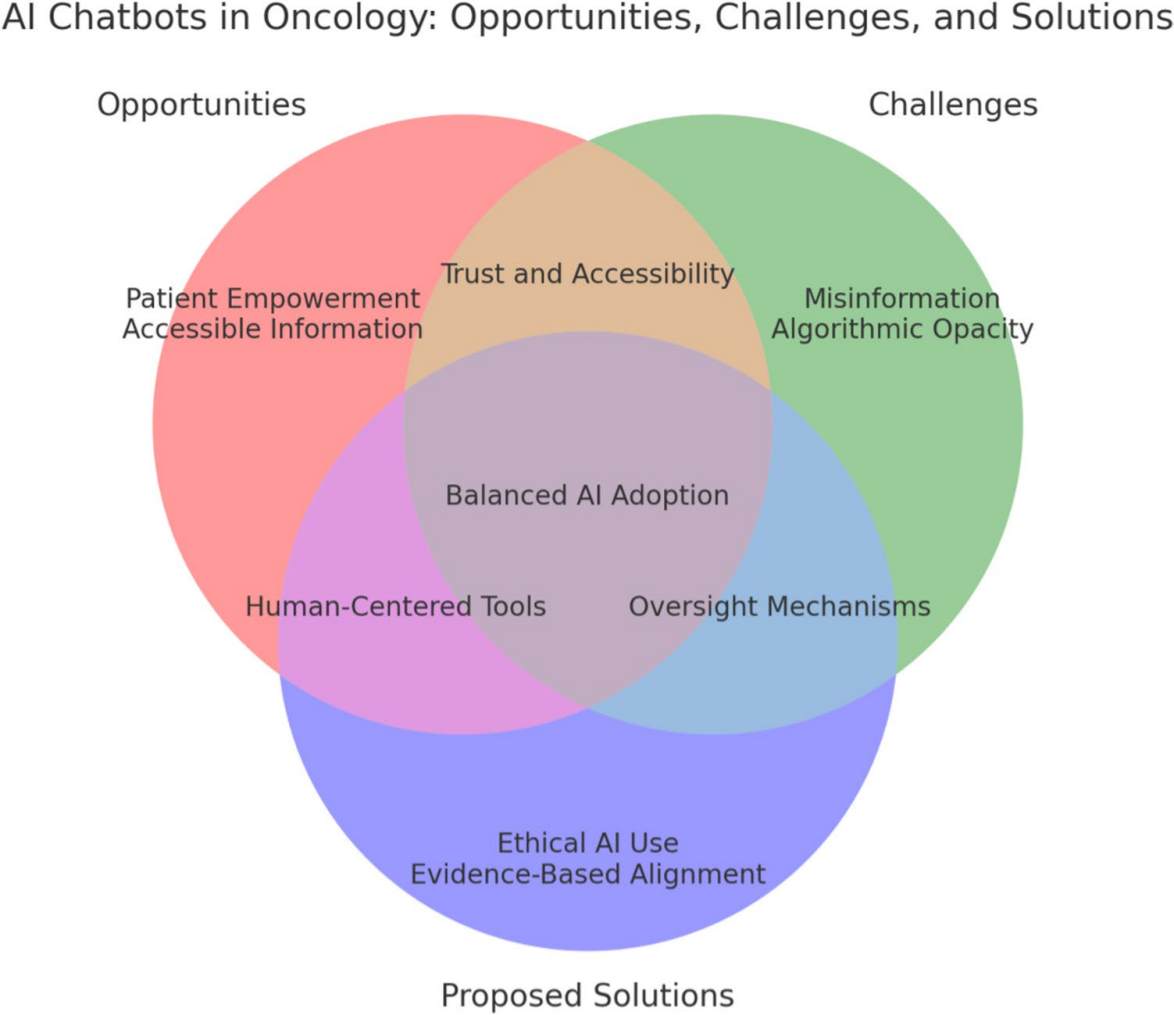
Balancing opportunities, challenges, and safeguards in AI chatbots for oncology. This Venn diagram illustrates the intersection of opportunities (e.g., patient empowerment, accessibility), challenges (e.g., misinformation risks, ethical concerns), and proposed solutions (e.g., evidence-based design, transparency measures) in the deployment of AI chatbots for oncology education. Overlaps emphasize areas requiring balanced approaches, such as trust building and safeguarding authenticity

A structured approach might begin with setting explicit boundaries (i.e., guardrails) regarding the chatbot’s scope: clarifying that it provides general educational pointers but not individual treatment prescriptions, and that no “emotional support” from the chatbot can replace real counseling from mental health professionals or oncology social workers [45]. As illustrated in Fig. 1, this involves balancing opportunities such as patient empowerment and accessibility with challenges like misinformation and ethical concerns, while integrating safeguards such as evidence-based design and transparency measures. After formal risk assessment, institutions could roll out pilot versions of the chatbot in controlled environments, collecting logs of user interactions and analyzing any recurring errors. The logs, stripped of identifiable data, would then be reviewed by certified oncologists and data ethics personnel, who would flag inaccuracies or manipulative language patterns. Only once the system meets stringent performance metrics, and has robust disclaimers integrated throughout its interface, would it be deployed more broadly [46].

Recent advancements have focused on developing robust safety guardrails for LLMs [47]. For instance, Meta’s Llama Guard system evaluates the user input, the LLM-generated output, and a predefined set of safety conditions to determine whether a response is “safe” or “unsafe.” However, a significant challenge lies in defining a comprehensive and effective list of safety conditions or examples. Moreover, research has demonstrated that these guardrails can sometimes be circumvented through sophisticated prompting techniques, highlighting the need for continuous refinement and improvement in safety mechanisms [48].

### Implications for Practice and the Role of Educators

While industry-led chatbot development marches forward, educators, professional societies, and policy-makers have critical roles to play in preventing an erosion of trust. From a pedagogical standpoint, one promising strategy involves equipping oncology trainees with skills to evaluate AI-based communication [49]. Such training would teach future clinicians how to identify chatbot inaccuracies, counsel patients about safe use of AI resources, and remain vigilant for manipulative or misleading chat partners. Another strategy might be to involve patient advocacy groups in chatbot oversight committees, ensuring that the voices of survivors and caregivers inform the design and maintenance of these systems.

Public outreach efforts could also educate patients on how to distinguish reliable digital tools from questionable ones. For example, public-awareness campaigns might highlight simple methods for verifying the chatbot’s affiliation (such as checking hospital or academic credentials), looking for standard disclaimers, and cross-referencing any critical recommendations with official cancer guidelines. This proactive approach might preserve the benefits of advanced AI, particularly the ability to disseminate health advice instantaneously, while curbing the illusions fostered by chatbots seeking to emulate supportive individuals.

### Envisioning the Future: A Cautious Yet Constructive Path

It would be a mistake to assume that all AI chatbots in oncology education are inherently harmful or that the “dead internet” scenario is uniformly bleak. On the contrary, well-designed AI systems could enhance knowledge dissemination, direct users toward credible sources, and alleviate some of the burden on oncology practitioners [50]. Yet the fundamental issues of misrepresentation and misguided trust, along with the broader backdrop of potential widespread bot proliferation, must be addressed head-on. Healthcare institutions, patient communities, and technology firms alike have a shared responsibility to enforce guardrails that minimize harm while preserving opportunities for constructive innovation.

A practical framework for addressing these challenges lies in the integration of academic rigor, transparent verification, and regular data updates. Technology providers could improve trust and reliability by making parts of their algorithmic architecture available for independent auditing and ensuring that training datasets are aligned with evidence-based cancer protocols. Such measures may help narrow the gap between the “human-like” responses generated by AI and the clinically grounded reality required for patient care. However, the prospect that a significant proportion of online cancer-related discussions may be artificially generated highlights the need for ongoing vigilance. This is particularly critical in psychosocial support, where the perceived authenticity of interactions plays a vital role in fostering trust, instilling hope, and providing meaningful emotional support.

Achieving a balanced way forward will rely on collaboration between all key parties. Oncologists and educators should critically review chatbot content to ensure it aligns with the latest research and professional guidelines. Regulatory bodies could introduce clear labeling requirements, ensuring AI tools transparently indicate their non-human status and provide clarity on how their reliability is judged. Tech platforms hosting these tools must adopt robust systems to swiftly address and correct any false or harmful information. In this way, AI’s role in oncology education can evolve into providing transparent, reliable, and ethically designed support, enhancing both learning and patient care without overstepping its limitations.

## Conclusion

AI chatbots offer a dual-edged potential in oncology education: they are powerful tools for delivering personalized, accessible cancer information but also pose significant risks of misinformation and user deception. Their advanced capabilities, driven by sophisticated language models, present unique opportunities for tailored education and support. However, this same technological sophistication raises concerns about the spread of unreliable information and the risk of users mistaking these tools for authoritative experts or empathetic peers. The emergence of human-like AI entities further highlights the need to address issues of authenticity, trust, and the integrity of online interactions, particularly in a field as sensitive as oncology.

To harness the benefits of AI while mitigating its risks, oncology stakeholders must implement context-specific safeguards that emphasize transparency, accountability, and adherence to evidence-based practices. Regulatory and professional frameworks should ensure that AI tools are clearly identified as non-human and their limitations explicitly communicated. By prioritizing these principles, the field can unlock the transformative potential of AI while remaining firmly committed to patient empowerment, trust, and human-centered care.

## Data Availability

No primary data were generated or analyzed in this narrative review. All data supporting the findings are derived from publicly available sources cited in the manuscript.
